# Diagnostic Efficacy of CT Examination on Early Detection of Lung Cancer during Pandemic of COVID-19

**DOI:** 10.3390/diagnostics12102317

**Published:** 2022-09-26

**Authors:** Yanjie Zhao, Ruibin Wang, Feng Shi, Jiangping Wu, Fusheng Jiang, Qingkun Song

**Affiliations:** 1 Department of Medical Oncology, Beijing Shijitan Hospital, Capital Medical University, Beijing 100038, China; 2 Department of Emergency, Beijing Shijitan Hospital, Capital Medical University, Beijing 100038, China; 3 Department of Pathology, Beijing Shijitan Hospital, Capital Medical University, Beijing 100038, China; 4 Department of Cancer Research, Beijing Shijitan Hospital, Capital Medical University, Beijing 100038, China; 5 Department of Thoracic Surgery, Beijing Shijitan Hospital, Capital Medical University, Tieyi Road 10, Haidian District, Beijing 100038, China; 6 Department of Clinical Epidemiology, Beijing Youan Hospital, Capital Medical Univerisity, Youanmenwai Xitoutiao 8, Fengtai District, Beijing 100069, China

**Keywords:** CT, lung cancer, squamous cell carcinoma, adenocarcinoma, TP53, Ki-67, non-smoking

## Abstract

Background: Since the outbreak of COVID-19 in 2020, routine CT examination was recommended to hospitalized patients at some hospitals and discovered lung cancer patients at an early stage. This study aimed to investigate the detection efficacy of routine CT examination on early diagnosis of lung cancer, especially on pathological characteristics. Methods: The epidemic of COVID-19 outbreak in January 2020 in China, and routine CT examination was recommended to hospitalized patients in June 2020 and ended in July 2021. Based on the time points, we compared the diagnosis efficacy between three periods: pre-period, peri-period, and the period of routine CT examination. Results: During the period of routine CT examination, more early stages of lung cancer were detected and the tumor size was reduced to 2.14 cm from 3.21 cm at pre-period (*p* = 0.03). The proportion of lung adenocarcinoma and early stage adenocarcinoma was increased by 12% and 30% in the period of routine CT examination, with referral to the pre-period of CT examination (*p* < 0.05). A total of 61% of diagnosed patients had the wild type of TP53 gene during the period of routine CT examination, compared to 45% of patients at the pre-period of CT examination (*p* = 0.001). The median Ki-67 index was 15% among patients diagnosed at the period of routine CT examination and increased to 35% at the pre-period of CT examination (*p* < 0.001). The period of routine CT examination was associated with a 78% higher probability of detecting an early stage of adenocarcinoma (OR = 1.78, 95%CI 1.03, 3.08) but no significant association was observed for squamous cell carcinoma. From the pre-period to the period of routine CT examination, the proportion of female patients and non-smoking patients increased by 57% and 44%, respectively (*p* < 0.001). Conclusion: Routine CT examination could detect more lung cancer at an early stage, especially for adenocarcinoma, and detect patients with less aggressive features. Further studies were warranted to confirm the findings.

## 1. Introduction

Lung cancer is the most common malignant carcinoma and ranks first in cancer-related mortality worldwide [1]. From 2007 to 2017, respiratory cancer cases increased by 37% [2]. China is also consistent with the world [3]. About 0.82 million patients were diagnosed with lung cancer in China in 2020 and increased by 6.5%, referring to that in 2018 [4], accounting for 37% of all new cases worldwide [1]. The mortality rate of lung cancer was higher in under-developed countries [1], and countries with a high sociodemographic index had 50% cancer cases but 30% deaths [2]. For the health inequities, cancer prevention and treatment is a priority for health investment [5], especially in under-developed countries. Screening and early diagnosis of lung cancer is an important measure to improve the survival rate and reduce the mortality rate [6]. Although there are many clinical studies on new biomarkers for lung cancer [7,8,9], the lack of screening programs was one of the main reasons for the challenging disease burden in developing countries [10].

The National Cancer Institute’s National Lung Screening Trial established that annual screening with low-dose computed tomography (LDCT) could reduce lung cancer mortality by 20% in specific high-risk individuals compared with X-ray screening [11]. In the US, the Prostate, Lung, Colorectal, and Ovarian (PLCO) study recommended X-ray scans not be used in screening programs for lung cancer [12]. LDCT was recommended for screening lung cancer [6]. In China, the definition of high-risk individuals for screening included age of 50–74 years, a 20 pack-year smoking history and smoking cessation within the past five years [13]. However, the uptake of LDCT has been quite low in the past few years. On 11 March 2020, the World Health Organization declared a pandemic outbreak of the novel coronavirus (COVID-19) [14], and the Beijing Municipal Health Commission initiated measures of COVID-19 prevention and control. For the detection of COVID-19 pneumonia, routine CT tests were subscribed to hospitalized patients at Beijing Shijitan Hospital from 17 June 2020 to 5 July 2021. In this study, we aimed to compare the efficacy of mandatory and opportunistic routine CT scans for the early detection of lung cancer. During the COVID-19 pandemic, there were many studies on the role of CT imaging in the diagnosis of lung diseases [15,16,17], but there are few studies on early screening of lung cancer.

## 2. Materials and Methods

### 2.1. Ethical Approval

All procedures performed in this study involving human participants were approved by the ethical committee of the Beijing Shijitan Hospital, Capital Medical University (Sjtky11-1x-2021(117)) on 8th December 2021, in accordance with the ethical standards of the 1964 Helsinki Declaration and its later amendments. Informed consent was obtained from the included subjects.

### 2.2. Study Settings

The study subjects were recruited from the Department of Thoracic Surgery, Beijing Shijitan Hospital, and Capital Medical University from January 2019 to July 2021. On 24 January 2020 National Health Commission announced the first level response to the outbreak of COVID-19. On 17 June, Beijing Hospitals Authority recommended routine CT examination to all hospitalized patients at outpatient service (mandatory CT examination) for the control of COVID-19, and this requirement ended on 5 July 2021. The routine CT was conducted without contrast agent and the scan parameters included: tube voltage of 80–120 kVp, tube current of 100–150 mAs, and scan pitch of 1.0. The CT scans were performed from the level of the apex to the base of the lungs bilaterally. After scanning, the raw data were transferred to the workstation for three-dimensional reconstruction with a layer thickness of 2 mm and a layer spacing of 2 mm. Based on the three time points, we defined the pre-period of CT examination ranging from January 2019 to January 2020 (opportunistic CT scan, they were evaluated for one or more of the symptoms of cough, dyspnea, chest pain, weight loss, and hemoptysis, or had a clinical suspicion of lung cancer), the peri-period of CT examination ranging from January 2020 to June 2020 (intermediate period of opportunistic and mandatory CT scan), and the period of routine CT examination ranging from June 2020 to July 2021 (mandatory CT scan). In the period of routine CT examination, all the hospitalized patients received routine CT tests at outpatient service and the patients with negative pneumonia reports, were eligible to be hospitalized.

### 2.3. Patients

The patients with suspected CT scan of lung cancer were recommended a pathological biopsy test. Patients with a pathological diagnosis of lung cancer were eligible to be included in the study and received thoracotomy surgery treatment.

### 2.4. Exposure and Definition

Age, sex, pathological type, American Joint Committee on Cancer (AJCC) stage, tumor size, and some biomarkers were collected and compared between the three periods. The pathological type was based on guidelines of the Chinese Society of Clinical Oncology 2021 [18]. Staging is based on the AJCC 8th Edition TNM lung cancer classification. Smoking history: “Never smoker” was defined as smoking less than 100 cigarettes or equivalent use of a pipe over a lifetime [19]. Family cancer history was considered as lung cancer occurrence in first-degree genetic relatives (parents, siblings, and offspring). Early detection of lung cancer was defined as the AJCC stage I.

### 2.5. Immunohistochemistry Test and Assessment

Expression of PD-L1, p53 protein (P53), and Ki-67 were detected by immunohistochemistry on 4 mm-thick formalin-fixed paraffin-embedded sections. Monoclonal antibodies against PD-L1 (rabbit anti-human, #SP-263), P53 (mouse anti-human, #DO-7), and Ki-67 (mouse anti-human, #MIB-1) were purchased from Beijing Zhong Shan Golden Bridge Biotechnology Co., Ltd. (Beijing, China).

Positive PD-L1 expression was characterized as brown cytomembrane staining. The expression rate of PD-L1 on tumor cells and immune cells was evaluated in all alive tumor cells and immune cells on the whole sections.

Positive P53 expression was characterized as nuclear staining. A mutant type of the TP53 gene was defined as negative P53 staining in tumor cell nuclei and >75% P53 staining in tumor cell nuclei. A wild type of the TP53 gene had ≤75% P53 staining in tumor cell nuclei.

Positive Ki-67 expression was characterized as nuclear staining. The areas with the highest numbers of Ki-67-labeled nuclei (hotspots) were used to determine Ki-67 expression under a low-power field. Then, 1000 cells were counted under a high-power field and the percentage of nuclear-positive cells was calculated as the Ki-67 index.

### 2.6. Statistical Analysis

All analyses were conducted in SPSS 23.0 software. Age, stage, tumor size, P53 expression, PD-L1 expression in immune cells, and Ki-67 index were analyzed by a Spearman correlation test with CT examination. Gender, pathological type, smoking history, family cancer history, and PD-L1 expression in tumor cells were analyzed by a Wilcoxon rank-sum test with CT examination. All the tests were two-sided, and the significant level was 0.05.

## 3. Results

At the pre-period of CT examination, 200 cases were diagnosed, 47 cases were diagnosed at the peri-period of CT examination, and 260 cases were diagnosed at the period of routine CT examination. The average age was 62.3 years old and 47% of the patients were female.

The proportion of lung cancer diagnosed at stage I was 47.0%, 63.8%, and 71.9% at the pre-period, the peri-period, and the period of routine CT examination, and increased by 53% (*p* < 0.001, Table 1). The pathological type of adenocarcinoma was 75% at the pre-period of CT examination and increased to 87% and 84% at the peri-period and the period of routine CT examination, respectively (*p* = 0.03, Table 1). The proportion of male patients was 65% at the pre-period of CT examination and decreased to 45% at the peri-period and routine CT examination (*p* < 0.001, Table 1).

**Figure 1:**
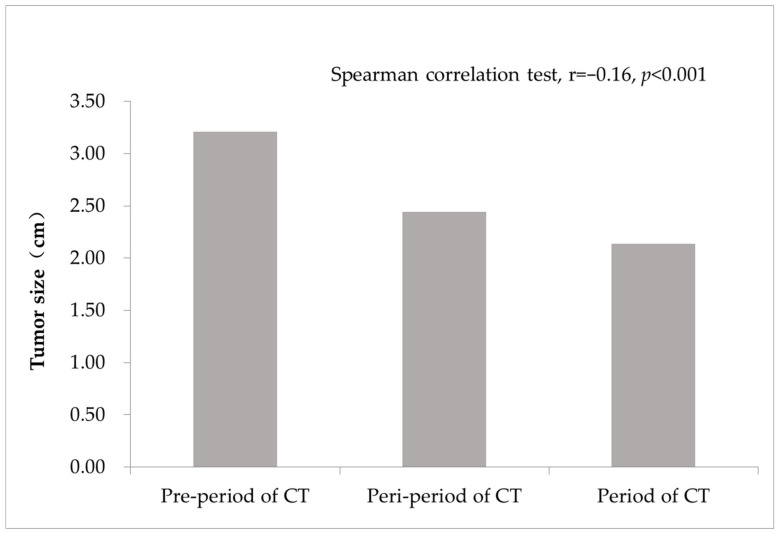
Tumor size of lung cancer diagnosed during the COVID-19 pandemic.

The average tumor size was 3.21 cm at the pre-period of CT examination, decreased to 2.44 cm at the peri-period of CT examination and 2.14 cm at the period of routine CT examination. The decrease trend was significant (Figure 1).

Among patients with adenocarcinoma, the proportion of stage I was 61% at the pre-period of CT examination and increased to 70% at the peri-period of CT examination and 79% at the period of routine CT examination (Table 2). The increasing trend was significant (*p* < 0.001). However, the patients with squamous cell carcinoma did not have any significant change in early detection (Table 2). Among the male patients, 37% were diagnosed at stage I at pre-period of CT examination and the proportion increased to 52% at the peri-period of CT examination and to 61% at the period of routine CT examination (*p* < 0.001, Table 2). The female patients also had a significant increase trend of early detection (Table 2). The proportion of stage I was 66% at the pre-period of CT examination and increased to 73% and 81% at the peri-period of CT examination and the period of routine CT examination (*p* = 0.005, Table 2).

Among all of the patients, 44% who were diagnosed at the pre-period of CT examination had a smoking history, and the figures reduced to 28% and 19% at the peri-period and the period of routine CT examination (Table 3). The trend of reduction was significant (*p* < 0.001). Family cancer history did not change during the three periods and more than 95% of patients reported negative history (Table 3).

Among the patients, 55% had a mutant type of TP53 gene at the pre-period of CT examination, and the proportions decreased to 50% and 40% at the peri-period of CT examination and the period of routine CT examination (*p* = 0.001, Table 4). The PD-L1 expression in tumor cells and immune cells did not change significantly with the three periods of CT examination (Table 4). The median Ki-67 index was 35%, 20%, and 15% in the pre-period, the peri-period, and the period of routine CT examination, and the decrease trend was significant (Table 4). The correlation coefficient was −0.22 between the Ki-67 index and the periods of CT examination (*p* < 0.001).

Routine CT examination was associated with a 90% higher probability of early detection of lung cancer (OR = 1.90) and for adenocarcinoma, the OR of early detection was 1.78 (95%CI 1.03, 3.08) (Table 5). For squamous cell carcinoma, the association was not significant (Table 5).

## 4. Discussion

Cancer has become a significant health, financial, and societal burden in China. Globocan 2020 reported that 23.7% of newly diagnosed cancer cases occurred in China, and lung cancer had the highest incidence [1]. LDCT is more effective than previous tools for diagnosing early lung cancer [20]. According to the results of the National Lung Cancer Screening Trial, LDCT screening was the most effective way to reduce lung cancer mortality [21]. However, in this study, we observed the significant effect of routine CT testing on early lung cancer among hospitalized patients, especially for adenocarcinoma. The detected cases were less aggressive and tended to have a higher proportion of the wild type of TP53 and a lower Ki-67 index.

National lung cancer screening guidelines recommend an annual LDCT examination focusing on high-risk individuals [13]. However, the Beijing Municipal Government decided to start the first level response to major public health emergencies at the outbreak of COVID-19 from 24 January 2020 [22]. The National Health Commission of the People’s Republic of China released the guidelines for the prevention and control of COVID-19 (the first edition) on 1 February 2020 [23]. In June 2020, Beijing reported the cluster cases of COVID-19, and the Beijing Hospitals Authority recommended all hospitalized patients undergo routine CT examination at an outpatient service from 17 June 2020, and this requirement ended on 5 July 2021. This decision provided an excellent chance to study the efficacy of routine CT examinations, including non-high-risk individuals of lung cancer. 

In our study, we found that the proportion of lung cancer patients diagnosed at stage I was increased and the average tumor size was decreased during the period of routine CT examination. Similar results were observed by the Cancer Hospital, Chinese Academy of Medical Sciences, that 76% of diagnosed patients were at stage I and II under LDCT screening [24].

Routine CT examinations detected more adenocarcinoma, more female patients, and more patients with a negative smoking history. Squamous cell carcinoma has a strong etiological association with cigarette smoking, whereas adenocarcinoma is the most common lung cancer type among non-smoking people [25]. At present, the definition of high-risk individuals in China [13] and the United States [11] focuses on people with a smoking history but not non-smoking people. This definition of high-risk individuals made it possible to miss patients with adenocarcinoma. Yang et al. [26] showed that the incidence rate of lung cancer among non-smoking people was significantly higher in China than in the United States, especially among women. They suggested that environmental air pollution exposure and gender should be included in the lung cancer risk prediction model to obtain a precise definition of high-risk individuals in China, especially among non-smoking women. More adenocarcinoma, more non-smoking patients, and more female patients diagnosed in routine CT examinations, indicated that non-smoking people, especially women, might also need CT screening. A randomized trial in the Netherlands also showed greater benefits for women than for men with CT screening [6].

More early stage adenocarcinoma was detected, but not squamous cell carcinoma in this study. From previous randomized controlled trial (RCT) studies [27,28,29,30], only high-risk individuals with a smoking history were included in screening programs, and the studies focused on morbidity and mortality, not the pathological type and stage. The pathological characteristics might be considered in further screening programs, and it may be meaningful to implement a precise screening program for people.

Ki-67 is identified as a nuclear non-histone protein [31] and expressed during all phases of the cell cycle except the resting stage (G0), it has been used as a marker to evaluate proliferation in NSCLC [32]. It is an indicator of malignant proliferative activity [33] and an independent predictor of malignance progression [33,34]. Our study found the median of the Ki-67 index was reduced in cases detected from the pre-period to the period of routine CT examination. These patients might have a better prognosis. The PD-L1 is variably expressed on the surface of cancer cells and antigen-presenting cells within tumor tissues, and indicates a potent inhibitory signal within the tumor microenvironment [35]. PD-L1 expression on tumor cells correlates with poor clinical prognosis in many cancers [36,37,38,39]. But Cooper et al. identified PD-L1 as a favorable prognostic factor in early stage NSCLC [40].

The p53 protein encoded by the wild type of TP53 gene is an important tumor suppressor protein in vivo, but the protein encoded by the mutant TP53 gene has a cancer-promoting effect and is associated with poor prognosis [41,42]. The TP53 gene can be used as an important indicator for prognosis assessment of lung cancer [43]. The immunohistochemistry test remains the most common choice to infer TP53 mutational status [44,45], and is an accurate surrogate for TP53 mutational analysis [44]. Our study found that the proportion of mutant type TP53 was reduced and the proportion of wild type TP53 was increased in the period of routine CT examination. Therefore, this result showed that the prognosis of lung cancer patients detected in the period of routine CT examination was better.

Routine CT examination increased early detection of lung cancer by 90%, especially adenocarcinoma (Table 5). This result further confirmed many previous studies [24,27,28,29,30], but the advantages in adenocarcinoma needed further verification. This result suggests that we might need to be concerned with the early detection of patients with adenocarcinoma. The early detection rate of lung adenocarcinoma was 4.8‰, 2.3‰, and 2.9‰ in the pre-period, the peri-period, and the period of routine CT examination. During the period of routine CT examination, on all hospitalized patients, including children, a CT scan was performed, therefore the lower early detection rate was non-comparable to that in the pre-period of routine CT examination. Further studies should focus on the target clinical patients for CT screening.

There were some limitations in our study. First, the sample size was small. Second, it was a single-center study and limited to hospitalized patients. Third, during the COVID-19 pandemic, the outpatients who were more likely to have concerning lung symptoms accessed CT scans more.

## 5. Conclusions

A routine CT examination was effective to detect the early stages of lung adenocarcinoma among hospitalized patients. The detected cases had less aggressive and better prognosis features. The definition of high-risk people for lung cancer screening programs might need to be reconsidered, especially for females and non-smokers. This study was significant for lung cancer screening programs, especially for the definition of high-risk people. Further studies are warranted to explore the target clinical patients for screening by routine CT scan.

## Figures and Tables

**Table 1:** Characteristics of lung cancer patients during pandemic of COVID-19.

	Pre-Period of CT (N = 200)	Peri-Period of CT (N = 47)	Period of CT (N = 260)	r/z Value	*p*
Stage				−0.24	<0.001
I	94 (47.0)	30 (63.8)	187 (71.9)		
II	24 (12.0)	7 (14.9)	20 (7.7)		
>II	82 (41.0)	10 (21.3)	53 (20.4)		
Pathological type				−2.17	0.030
Adenocarcinoma	135 (75.0)	40 (87.0)	202 (83.8)		
Squamous cell carcinoma	45 (25.0)	6 (13.0)	39 (16.2)		
Age (mean ± SD)	62.0 ± 10.34	61.0 ± 8.90	62.8 ± 11.61	0.05	0.234
Gender				−4.24	<0.001
Male	130 (65.0)	21 (44.7)	116 (44.6)		
Female	70 (35.0)	26 (55.3)	144 (55.4)		

**Table 2:** Effect of routine CT examination on early diagnosis of lung cancer.

Stage		Pre-Period of CT, n(%)	Peri-Period of CT, n(%)	Period of CT, n(%)	*p*
Adenocarcinoma (N = 377)	I	82 (60.7)	28 (70.0)	160 (79.2)	<0.001
	II	11 (8.1)	4 (10.0)	11 (5.4)	
	>II	42 (31.1)	8 (20.0)	31 (15.3)	
Squamous cell carcinoma (N = 90)	I	9 (20.0)	2 (33.3)	13 (33.3)	
	II	10 (22.2)	2 (33.3)	8 (20.5)	0.176
	>II	26 (57.8)	2 (33.3)	18 (46.2)	
Male (N = 267)	I	48 (36.9)	11 (52.4)	71 (61.2)	
	II	22 (16.9)	3 (14.3)	11 (9.5)	<0.001
	>II	60 (46.2)	7 (33.3)	34 (29.3)	
Female (N = 240)	I	46 (65.7)	19 (73.1)	116 (80.6)	
	II	2 (2.9)	4 (15.4)	9 (6.3)	0.005
	>II	22 (31.4)	3 (11.5)	19 (13.2)	

**Table 3:** Distribution of risk factors among lung cancer patients.

	Pre-Period of CT	Peri-Period of CT	Period of CT	*p*
Smoking history				<0.001
Negative	113 (56.5)	34 (72.4)	211 (81.2)	
Positive	87 (43.5)	13 (27.7)	49 (18.8)	
Family cancer history				0.719
Negative	191 (95.5)	46 (97.9)	251 (96.5)	
Positive	9 (4.5)	1 (2.1)	9 (3.5)	

**Table 4:** The biomarker of lung cancer changed with CT examination.

	Pre-Period of CT	Peri-Period of CT	Period of CT	*p*
TP53				0.001
Mutant type	102 (54.8)	22 (50.0)	94 (39.5)	
Wild type	84 (45.2)	22 (50.0)	144 (60.5)	
PD-L1 in tumor cells				0.240
<1%	96 (52.5)	27 (62.8)	141 (59.7)	
≥1%	87 (47.5)	16 (37.2)	95 (40.3)	
PD-L1 in immune cells				0.081
<1%	41 (24.3)	11 (26.2)	32 (13.6)	
<5%	44 (26.0)	6 (14.3)	74 (31.5)	
<10%	44 (26.0)	13 (31.0)	64 (27.2)	
≥10%	40 (23.7)	12 (28.6)	65 (27.7)	
Ki-67, median (IQR)	35 (60)	20 (50)	15 (35)	<0.001

**Table 5:** Multivariate analysis for early detection of lung cancer.

		Crude OR (95%CI)	*p*	Adjusted OR (95%CI) *	*p*
All patients	Pre-period of CT	1.00		1.00	
	Peri-period of CT	1.99 (1.03, 3.84)	0.040	1.41 (0.67, 2.95)	0.368
	Period of CT	2.89 (1.96, 4.26)	<0.001	1.90 (1.20, 2.99)	0.006
Adenocarcinoma	Pre-period of CT	1.00		1.00	
	Peri-period of CT	1.51 (0.71, 3.22)	0.289	1.21 (0.53, 2.75)	0.651
	Period of CT	2.46 (1.52, 4.00)	<0.001	1.78 (1.03, 3.08)	
Squamous cell carcinoma	Pre-period of CT	1.00		1.00	
	Peri-period of CT	2.00 (0.32, 12.69)	0.462	1.44 (0.18, 11.51)	0.731
	Period of CT	2.00 (0.74, 5.37)	0.169	1.41 (0.47, 4.23)	0.540

* Adjusting age, sex, pathological type, smoking history, TP53 genotype and Ki-67 index.

## Data Availability

The datasets generated during and/or analyzed during the current study are not publicly available but are available from the corresponding author on reasonable request.
